# Tumour acidosis evaluated in vivo by MRI-CEST pH imaging reveals breast cancer metastatic potential

**DOI:** 10.1038/s41416-020-01173-0

**Published:** 2020-12-01

**Authors:** Annasofia Anemone, Lorena Consolino, Laura Conti, Pietro Irrera, Myriam Y. Hsu, Daisy Villano, Walter Dastrù, Paolo E. Porporato, Federica Cavallo, Dario Livio Longo

**Affiliations:** 1 Department of Molecular Biotechnology and Health Sciences, Molecular Imaging Center, University of Torino, Via Nizza 52, Torino, Italy; 2 Department of Molecular Biotechnology and Health Sciences, University of Torino, Via Nizza 52, Torino, Italy; 3 University of Campania “Luigi Vanvitelli”, Viale Abramo Lincoln, 5, Caserta, Italy; 4 Institute of Biostructures and Bioimaging (IBB), Italian National Research Council (CNR), Via Nizza 52, Torino, Italy

**Keywords:** Cancer microenvironment, Cancer imaging, Breast cancer

## Abstract

**Background:**

Tumour acidosis is considered to play a central role in promoting cancer invasion and migration, but few studies have investigated in vivo how tumour pH correlates with cancer invasion. This study aims to determine in vivo whether tumour acidity is associated with cancer metastatic potential.

**Methods:**

Breast cancer cell lines with different metastatic potentials have been characterised for several markers of aggressiveness and invasiveness. Murine tumour models have been developed and assessed for lung metastases and tumour acidosis has been assessed in vivo by a magnetic resonance imaging-based chemical exchange saturation transfer (CEST) pH imaging approach.

**Results:**

The higher metastatic potential of 4T1 and TS/A primary tumours, in comparison to the less aggressive TUBO and BALB-neuT ones, was confirmed by the highest expression of cancer cell stem markers (CD44^+^CD24^−^), highlighting their propensity to migrate and invade, coinciding with the measurement obtained by in vitro assays. MRI-CEST pH imaging successfully discriminated the more aggressive 4T1 and TS/A tumours that displayed a more acidic pH. Moreover, the observed higher tumour acidity was significantly correlated with an increased number of lung metastases.

**Conclusions:**

The findings of this study indicate that the extracellular acidification is associated with the metastatic potential.

## Background

Breast cancer is the second leading cause of cancer-related death in women worldwide. In these patients, metastases at distant sites and not the primary tumours are still the main cause of death.^1^ In the past years, the rates of metastasis and mortality in breast cancer patients have decreased as a result of increased screening procedures and early diagnosis provided by several imaging techniques (i.e. mammography, ultrasonography and magnetic resonance imaging (MRI)). Unfortunately, by the time many breast tumours are detected with such techniques, metastasis may have already begun and remains unnoticed on imaging. Even at the time of initial cancer surgery, metastasis may have already occurred in many patients who eventually die of breast cancer.^2^ Once the tumour has spread, fewer curative treatment options are available to reduce cancer mortality upon metastasis.^3^ Predicting the tendency of a primary tumour to form secondary metastatic lesions, or its metastatic potential, remains a major challenge in cancer research. In fact, knowledge of tumour metastatic potential may help physicians to select the adequate treatment based on tumour aggressiveness while avoiding unnecessary side effects on patients.^4^ Established breast cancer prognostic markers are based on size and grade of the primary tumour and on the presence of lymph-node metastasis.^5^ However, definitive diagnosis and treatment planning are currently based on biopsy samples that can assess only limited portions of the tumour tissue with limited success in assessing its heterogeneity. Therefore, histological typing is only a weak prognostic marker of metastasis risk.

Indeed, tumour aggressiveness is associated with many processes, including hypoxia, altered glucose metabolism, angiogenesis and inflammation that determine the heterogeneity of tumour microenvironment.^6,7^ Among the several factors, the compensatory hypoxia-induced shift to anaerobic glycolysis for energy production can lead to a significant increase in production and release of acidic metabolites into the extracellular matrix (ECM). As a consequence, the progressive reduction of extracellular pH (pHe) within tumours, known as acidosis, is another feature of the tumour microenvironment.^8^ Tumour acidity has shown to affect most of the steps in the metastatic cascade, either directly or indirectly. In fact, acidic microenvironment is closely associated with increased mutagenesis, sustained proliferation and drug resistance, leading to more aggressive behaviour and metastasis.^9–11^

To date, the various steps of the metastatic cascade have been investigated by imaging-based approaches, looking for relationships between cancer aggressiveness and tumour vascularisation,^12^ hypoxia,^13^ redox status,^14^ metabolites and several other targets of the tumour microenvironment.^15–19^

On the other hand, very few studies have investigated tumour acidosis as a possible marker of the metastatic potential. One reason relies in the poor spatial resolution, long acquisition times, limited accuracy and reduced clinical translatability of the proposed approaches for measuring pH.^20^ Recently, MRI-based approaches exploiting the pH-sensitive chemical exchange-dependent saturation transfer (CEST) technique have been successfully investigated and validated for measuring tumour pH.^21^ Among these approaches, MRI-CEST pH mapping exploiting x-ray contrast agents have been shown to provide accurate pH measurements with high spatial resolution.^22–24^ Such improvements allowed for the first time to investigate the relationship between tumour metabolism and acidosis, providing evidence of their relationship in a breast murine model.^25^ In addition, the capability to quantify the heterogeneity of tumour pHe has been exploited for assessing the early therapeutic effect of drugs targeting tumour metabolism and for monitoring the onset of resistance.^26^ Other studies have explored tumour pH imaging for evaluating treatment response to other drugs affecting various pathways of tumour metabolism.^27–29^ Moreover, these molecules have also been validated for characterising tumour perfusion properties.^30,31^ Notably, this approach, owing to the clinical safety of the iodinated contrast agents, has already been translated to the clinic providing tumour pHe maps in breast and ovarian cancer patients.^32^ Therefore, MRI-CEST pH imaging holds promise to quantitatively assess tumour pHe with high accuracy and spatial resolution and our hypothesis is that this approach can provide reliable information on the role of tumour acidosis in cancer progression and metastasis.

In this study, we investigated several murine breast cancer models from low to highly metastatic that showed different cancer aggressiveness and invasiveness both in vitro and in vivo. Using MRI-CEST pH imaging, we observed that more aggressive cancer murine models showed increased tumour acidity in comparison to less aggressive ones.

## Methods

### Cell culture

TUBO cells were derived from a lobular carcinoma arising spontaneously in a BALB-neuT mouse^33^ and were grown in Dulbecco’s modified Eagle’s medium supplemented with 20% foetal bovine serum (FBS) and 100 U/mL penicillin and 100 µg/mL streptomycin (Pen/Strep); 4T1 and TS/A cells were grown in RPMI-1640 medium supplemented with 10% FBS, 100 U/mL Pen/Strep and 2 mM l-glutamine. TUBO is considered to be a non-metastatic cell line; triple-negative breast cancer 4T1 cells, instead, are a highly metastatic cell line derived from a spontaneous BALB/c mammary tumour^34^ and HER2^+^ TS/A cells are a metastasising cell line derived from a spontaneous BALB/c mammary tumour.^35^ Cells were not authenticated but tested as mycoplasma-free and passaged in our laboratory for <2 months after their resuscitation.

4T1 chronically exposed to acidic pH (pH 6.8) were maintained in RPMI-1640 supplemented with 10% FBS, 100 U/mL Pen/Strep and 2 mM l-glutamine and with 25 mmol/L of PIPES and HEPES to adjust pH to 6.8.^36^ We maintained tumour cells in this medium for 8 to 10 weeks until they completely recovered and grew at the same rate as parental cells.

### Western blotting and real-time PCR

Protein extraction was performed as previously described,^37^ and protein contents were quantified by BCA assay according to the manufacturer’s instructions (Thermo Fisher 23221). For western blotting, 20 µg were loaded per well on 7.5% precast TGX gel (Bio-Rad 4561026). Proteins were then transferred on PVDF membrane (Bio-Rad 10026933). The primary antibodies used for western blotting were as follows: CA IX (Novus Biological, 1:1000), NHE1 (Novus Biological, 1:1000) and vinculin (Santa Cruz Biotechnology 73614, 1:5000). Secondary antibodies were purchased from Sigma (anti-mouse IgG: A4416; anti-rabbit IgG: A6154). Clarity-ECL substrate was used to reveal bands (Bio-Rad 1705060) and protein expression was finally determined by quantification of band intensity using ImageLab (Bio-Rad). Real-time PCR method is described in Supplementary Material.

### pHe measurements

A total of 5 × 10^4^ TUBO, TS/A or 4T1 cells were seeded into 96-well plates and incubated overnight to allow adherence. The following day, the pHe was measured using a pH-sensitive fluorescent probes (pH-Xtra, Luxcel Bioscience, Cork, Ireland), as described in Supplementary Material.^38^

### Cell migration and invasion assays

For migration and invasion assays, cells were plated and carried out and analysed as described in Supplementary Material.

### Animal studies and MRI-CEST pH imaging

BALB/c mice were purchased from Charles River Laboratories (Calco, Italy), while BALB-neuT mice^39^ were bred at the Department of Molecular Biotechnology and Health Sciences, University of Torino, Italy. Mice were maintained in specific pathogen-free conditions (Allentown Caging Equipment, Allentown Inc.) and treated in accordance with University Ethical Committee and European guidelines under directive 2010/63. During housing, animals were monitored twice daily for health status. BALB/c mice (8–10 weeks old, mean weight 22 g) were inoculated subcutaneously (s.c.) with TUBO (1 × 10^5^ cells, *n* = 8), 4T1 (3 × 10^4^ cells, *n* = 11) and TS/A (2.5 × 10^5^ cells, *n* = 12) cells on both flanks. BALB-neuT mice (*n* = 8) were selected for the study from 25 weeks of age (mean weight 25 g). Altogether, pHe maps were measured for 22, 24, 16 and 16 4T1, TS/A, TUBO and BALB-neuT tumours, respectively, and tumour volumes ranged between 100 and 500 mm^3^.

4T1 pH 6.8 and 4T1 tumour-bearing animals (3 × 10^4^ 4T1 pH 6.8 and 3 × 10^4^ 4T1 cells subcutaneously injected, *n* = 10 for each group) 6 days following tumour inoculation were divided into two groups: one (control) was provided with drinking water and the other (bicarbonate) was provided with 200 mmol/L NaHCO_3_ ad libitum, which continued for the duration of the experiment. 4T1 pH 6.8 and 4T1 tumour-bearing mice were imaged for tumour pH measurement 7 days after treatment and mice group were treated until tumour volumes reached 400 mm^3^.

Prior to starting MRI acquisition, mice were anesthetised by injecting a mixture of xylazine 5 mg/kg (Rompun, Bayer, Milan, Italy) and tiletamine/zolazepam 20 mg/kg (Zoletil 100, Virbac, Milan, Italy) and breath rate was monitored by an air pillow placed below the animal (SA Instruments, Stony Brook, NY, USA) during all the experiment. MRI-CEST pH mapping was performed upon intravenous injection of ca. 270 µL of iopamidol (dose: 4 g iodine/kg body weight, Bracco Imaging SpA, Colleretto Giacosa, Italy) into the tail vein through a 27-gauge needle catheter. MRI-CEST images were acquired on a Bruker Avance 7 T MRI scanner using a 30-mm insert coil. A detailed description of the acquisition protocols is provided in Supplementary Material.

The acidity score was calculated to assess the heterogeneous pHe distribution within the tumour regions.^26^ Tumour pixels were clustered into three groups: group I for pixels displaying pHe values >7.0, group II for pixels displaying pHe values >6.7 and <7.0 and group III for pixels displaying pHe values <6.7. The percentage of pixels of each group was multiplied by a factor between 1 and 3, to obtain the acidity score, in accordance with the equation:

$${\mathrm{Acidity}}\;{\mathrm{score}} =	 \, \{\left[ {1 \times (\% {\mathrm{of}}\;{\mathrm{pixels}}\;{\mathrm{with}}\;{\mathrm{pHe}} \, > \, 7.0)} \right] \\ 	 + \left[ {2 \times (\% {\mathrm{pixels}}\;{\mathrm{with}}\;6.7 \, < \, {\mathrm{pHe}} \, < \, 7.0)} \right] \\ 	 + \left[ {3 \times (\%\;{\mathrm{pixels}}\;{\mathrm{with}}\;{\mathrm{pHe}} \, < \, 6.7)} \right] \}.$$

The acidity score ranges from 1 (less acidic) to 3 (more acidic), defining tumour regions with different acidosis levels.

To collect tumours, mice were sacrificed by cervical dislocation right after the end of the acquisition.

### FACS analysis

For flow cytometry-based glucose uptake assays, 4 × 10^5^ cells were incubated with 100 μM 2-NBDG (2-deoxy-2-[(7-nitro-2,1,3-benzoxadiazol-4-yl) amino]-d-glucose, Focus Biomolecules, Plymouth, USA) dissolved in glucose-free medium for 10 min, and their fluorescence was measured by flow cytometry as described in Supplementary Material.

Moreover, cells and fresh primary tumours were stained for membrane antigens. The following antibodies were used: Alexa Fluor647-conjugated anti-stem cell antigen-1 (Sca1), phycoerythrin (PE)-conjugated anti-CD44 and PE/Cy7-conjugated anti-CD24 (all from BioLegend). All samples were collected and analysed using a CyAn ADP Flow Cytometer and Summit 4.3 software (DakoCytomation) as described in detail in Supplementary Material.

### Immunofluorescence microscopy

Fixed cells and frozen tumour samples were stained for GLUT1. See Supplementary Material for further details.

### Histologic assay for lung metastasis

Spontaneous pulmonary metastases were studied by histological examination in tumour-bearing mice when the primary tumours had grown to a volume of ca. 400 mm^3^ (ca. 10 mm diameter) (*n* = 5 mice for each breast cancer murine model). Immediately afterwards, mice were euthanised, and resected lungs were fixed in phosphate-buffered 4% paraformaldehyde and paraffin-embedded. Histological sections were cut from each lobe at 100 μm intervals and stained with haematoxylin and eosin. Metastases were counted with a Nikon SMZ1000 stereomicroscope (Nikon, Amsterdam, The Netherlands) and ImageJ software (US National Institutes of Health, Bethesda, MD, USA).^40^ Groups of five or more tumour cells were scored as a metastasis. Moreover, the percentage of the area occupied by metastases was calculated by measuring the area of metastasis in respect to the total lung area by using ImageJ.

### Statistical analysis

Statistical analysis was performed by using the GraphPad Prism 7 software (GraphPad Inc., San Diego, CA, USA). Data are presented as mean ± SD, unless otherwise stated. One-way analysis of variance (ANOVA) and Bonferroni’s multiple comparison test were used to test for statistically significant differences between the measurements of parameters among the four groups of breast cancers. The Pearson’s product–moment correlation test was used to search for correlation between parameters. For all tests, a *P* value < 0.05 was considered statistically significant.

## Results

### The in vitro aggressiveness and migration capability of breast cancer cells correlates with the expression of specific markers of the metastatic potential

We evaluated the invasiveness and migration capability of some murine breast cancer cell lines and whether their aggressiveness was related to specific molecular markers.

**Fig. 1:**
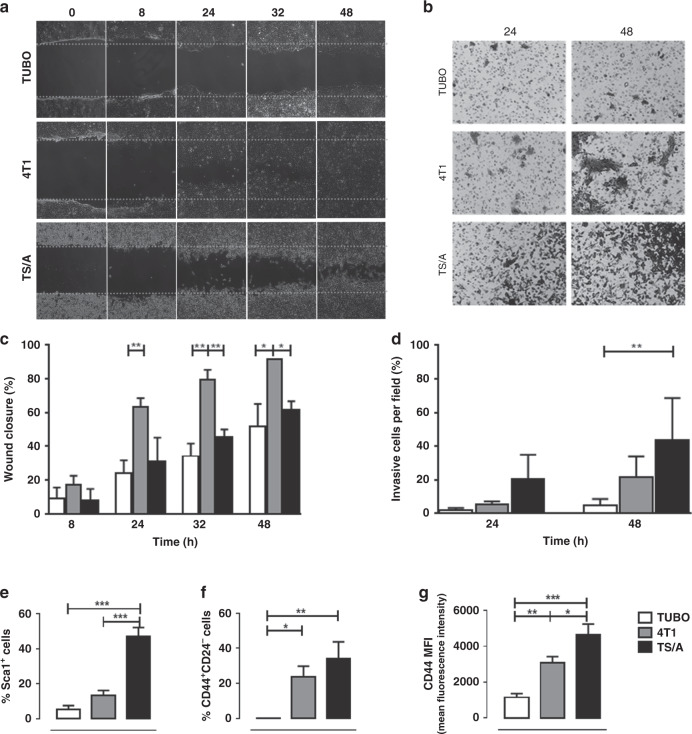
Characterisation of migration and invasion capability in vitro of TUBO, 4T1 and TS/A cells. **a** Representative images of cell migration and wound repair; cells were allowed to migrate 8, 24, 32 and 48 h after wounding. **b** Representative images of cell invasion; cells were allowed to invade the Matrigel-coated transwell membrane for 24 and 48 h. **c** TUBO cell migration and wound repair capability was slower compared to 4T1 cells; data are shown as mean ± SD (*n* = 3). *P < 0.05; **P < 0.01. **d** TS/A cells were the most invasive cells after 24 and 48 h; data are shown as mean ± SD (*n* = 3). **P < 0.01. **e**–**g** Graphs showing the mean ± SD of Sca1^+^, CD44^+^CD24^−^ cells and CD44 mean fluorescence intensity (MFI) from three independent experiments. *P < 0.05; **P < 0.01; ***P < 0.001.

In vitro cell migration studies showed that TUBO cells were slower to close the wound compared to 4T1 and TS/A cells (Fig. 1a). 4T1 cells exhibited a significantly faster rate of wound closure than TS/A and TUBO cells (wound closure of 63%, 31%, 24% after 24 h for 4T1, TS/A and TUBO cells, respectively, *P* < 0.01, Fig. 1c). These data suggest that 4T1 cells have a more rapid migration than TS/A and TUBO cells. Furthermore, cell invasion assays showed that the invasion capability followed the order TS/A > 4T1 > TUBO cells both after 24 and 48 h (Fig. 1b). After 48 h of incubation, TUBO cells exhibited the lowest invasiveness compared to 4T1 and TS/A cells (almost 5% of invasiveness after 48 h, Fig. 1d).

To further investigate the metastatic potential of the different cell lines in vitro, we analysed by flow cytometry the presence of cancer stem cells (CSCs), a cell population characterised by self-renewal and tumour-forming abilities, thereby promoting tumour growth and metastases formation. As shown in Fig. 1e, f, the percentage of cells expressing the CSC marker Sca1^40^ and of CD44^+^CD24^−^ cells^41^ was low in TUBO, but intermediate in 4T1 and high in TS/A cells, indicating that higher levels of CSCs were correlated with the migration and invasion capabilities of breast cancer cells. Moreover, the expression of CD44, a cell surface marker whose activation by its ligand hyaluronan induces cytoskeletal changes, promotes cell proliferation and survival and enhances cellular motility, thus contributing to the metastatic process,^42^ progressively increased from TUBO to 4T1 and TS/A cells (Fig. 1g and Supplementary Fig. S1). Collectively, these data confirmed that breast cancer cells expressing CD44^+^CD24^−^ phenotype have enhanced invasive properties.

### The pHe of breast cancer cell lines is associated with glucose uptake and invasiveness

**Fig. 2:**
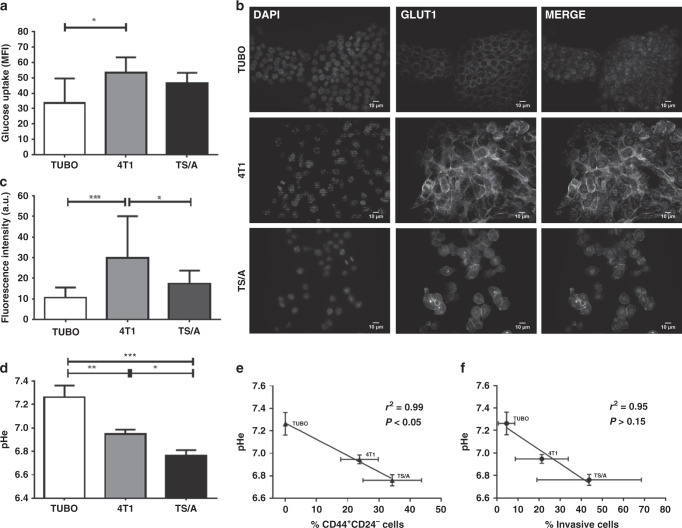
Metabolic characterisation of TUBO, 4T1 and TS/A cells. **a** Glucose uptake in TUBO, 4T1 and TS/A cells. Results are expressed as mean ± SD (*n* = 3). *P < 0.05. **b** Immunofluorescence labelling of GLUT1 in TUBO, 4T1 and TS/A cells. Channel intensities are identical across each line for DAPI or GLUT1 signal, white bar (10 µm). **c** Fluorescence intensity of GLUT1 expression in TUBO, 4T1 and TS/A cells. *P < 0.05; ***P < 0.001. **d** Extracellular pH measurement, *P < 0.05; **P < 0.01; ***P < 0.001 (*n* = 3). **e**, **f** Correlation between in vitro extracellular pH measurement and CD44^+^CD24^−^ cells or percentage of invasive cells (*r*^2^ = 0.99, *P* < 0.05 and *r*^2^ = 0.95, *P* > 0.15, respectively).

High metabolic rates in cancer cells determine the net production of protons that are secreted for maintaining the intracellular pH homeostasis. Therefore, a complex interplay between glycolytic activity, glucose transporters and acid–base transporters results in the net extracellular acidification that favours cell migration and invasion. By means of a fluorescent glucose analogue (2-NBDG, Fig. 2a and Supplementary Fig. S2), cancer cell glucose uptake was analysed and a marked uptake was observed for 4T1 and TS/A cells in comparison to TUBO ones (MFI 34 ± 16, 53 ± 10 and 46 ± 7 for TUBO, 4T1 and TS/A, respectively), suggesting that TS/A and 4T1 are more avid for glucose. This was also confirmed by the higher expression of GLUT1 transporter on the cell membrane observed in 4T1 and TS/A cell lines compared to TUBO cells using immunofluorescence staining (mean fluorescence intensity of 30 ± 20, 17 ± 6 and 11 ± 5 for 4T1, TS/A and TUBO, respectively, Fig. 2b, c).

As a consequence of the increased glucose uptake, we may expect differences in the acidification rate of the extracellular space for the three cell lines. Compared to TS/A and 4T1, the extracellular space in TUBO cells was significantly less acidic, as indicated by a higher pHe (*P* = 0.0003, pHe mean value 7.26, 6.76 and 6.94 for TUBO, 4T1 and TS/A, respectively, Fig. 2d). Interestingly, we found a significant inverse correlation between pHe and CD44^+^/CD24^−^ cell phenotype (*P* < 0.05, *r*^2^ = 0.99, Fig. 2e) and a strong, but not significant inverse correlation between pHe and the percentage of invasive cells (*P* > 0.15, *r*^2^ = 0.95, Fig. 2f). Intriguingly, glycolytic cells presented lower protein levels of CA9 and NHE1, as opposed to the minimally glycolytic TUBO cells^43^ (Supplementary Fig. S3a, b). On the other hand, when compared to TUBO cells, both 4T1 and TS/A cell lines showed similar expression levels of MCT4, but higher levels of MCT1 (Supplementary Fig. S3c, d), which has been shown to mediate acid efflux. Our findings collectively suggest that MCT1 is more involved in the net acid efflux of TS/A and 4T1 cells, although several other acid-based transporters might contribute.

### In vivo development of lung metastasis is compared to glucose uptake and to CSC markers

**Fig. 3:**
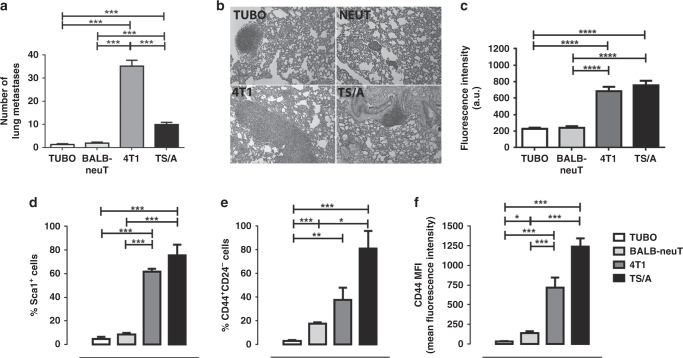
4T1 developed a higher number of lung metastases in vivo. **a** Lung metastases count. ***P < 0.001. **b** Representative H&E staining of lung metastases of BALB/c mice s.c injected with TUBO, 4T1 or TS/A cells and BALB-neuT mice. Amplification ×10. **c** GLUT1 fluorescence intensity calculated for BALB-neuT, 4T1 and TS/A tumour slice compared to TUBO intensity. ****P < 0.0001. **d**–**f** Cytofluorimetric analysis of 10 mm mean diameter tumours (400 mm^3^) explanted from BALB-neuT mice or from BALB/c s.c. injected with TUBO, 4T1 or TS/A cells. Graphs showing the mean ± SD of the percentage of Sca1^+^ or CD44^+^CD24^−^ cells and CD44 mean fluorescence intensity (MFI) from three independent experiments. *P < 0.05; **P < 0.01; ***P < 0.001.

To study the impact of the different metastatic potential on the formation of lung metastases, BALB/c female mice were injected s.c. in the flank with TUBO, 4T1 or TS/A cells. These mice and transgenic BALB-neuT female mice, which spontaneously develops autochthonous mammary carcinomas, were culled when their primary tumours reached a volume of ca. 400 mm^3^, lungs were excised and the number of metastases was quantified histologically. As reported in the representative images, mice s.c. injected with 4T1 cells showed larger lung metastasis and the highest number of spontaneous lung metastases (mean value = 35.0 ± 11.0) compared to the other breast cancer models used (Fig. 3a, b). The number of metastatic nodules slightly decreased in mice injected with TS/A (mean value = 10 ± 3.8), whereas the metastases were barely detectable in the lungs of BALB-neuT mice (mean value = 1.9 ± 2.2), and were even less in mice injected with TUBO cells (mean value = 1.3 ± 1.5). Also, when considering the area of the lungs occupied by metastases, both 4T1 and TS/A tumours showed significant greater values (Supplementary Fig. S4).

To determine the importance of the glucose consumption in the observed extracellular acidification, breast tumours were sectioned and stained for GLUT1 (Supplementary Fig. S5). TUBO and BALB-neuT tumours expressed a lower level of GLUT1 as compared to 4T1 and TS/A (fluorescence intensity, 239.8 ± 20.6, 229.8 ± 12.43, 685.3 ± 53.3 and 756.9 ± 55.42 for BALB-NeuT, TUBO, 4T1 and TS/A tumours, Fig. 3c), confirming that increased glucose demand is associated to more aggressive tumours, as observed for the cell lines in vitro.

We next asked whether inherently different tumour aggressiveness between the four breast murine models might be explained by a different expression of CSC markers. Cytofluorimetric analysis of Sca1^+^ and CD44^+^/CD24^−^ was performed in the primary tumours explanted from BALB-neuT mice or from BALB/c mice s.c. injected with TUBO, 4T1 or TS/A cells. As shown in Fig. 3d and Supplementary Fig. S6, TUBO and BALB-neuT tumours displayed a very low percentage of Sca1^+^ CSCs, which were significantly higher in both 4T1 and TS/A tumours. Similarly, CD44^+^/CD24^−^ CSCs were present at low frequency in TUBO tumours and slightly increased in BALB-neuT tumours, while they were abundant in both 4T1 and TS/A tumours (Fig. 3e), in agreement with the results obtained in vitro. Moreover, the expression level of CD44 was low in TUBO and BALB-neuT tumours and significantly increased in 4T1 and TS/A tumours (Fig. 3f and Supplementary Fig. S6). Altogether, these results confirm the different metastatic potential of the investigated breast cancer models, with 4T1 and TS/A being the more aggressive cell lines both in vitro and in vivo.

### Invasive breast tumour murine models showed an increased tumour acidosis

**Fig. 4:**
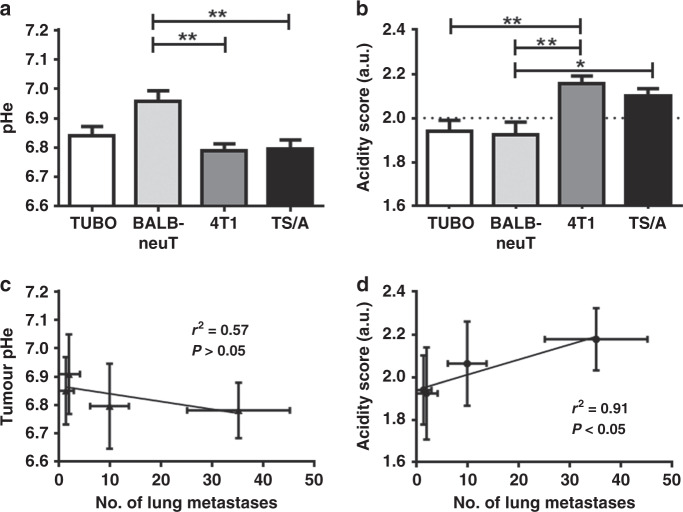
Tumour pHe correlates in vivo with number of lung metastasis. **a** Mean extracellular pH measured in TUBO, BALB-neuT, 4T1 and TS/A tumour. **P < 0.01. **b** Acidity score calculated for TUBO, BALB-neuT, 4T1 and TS/A tumour. *P < 0.05 and **P < 0.01. **c** Correlation between tumour pHe and number of lung metastases (*r*^2^ = 0.57, *P* > 0.05). **d** Correlation between acidity score and number of lung metastases (*r*^2^ = 0.91, *P < 0.05).

**Fig. 5:**
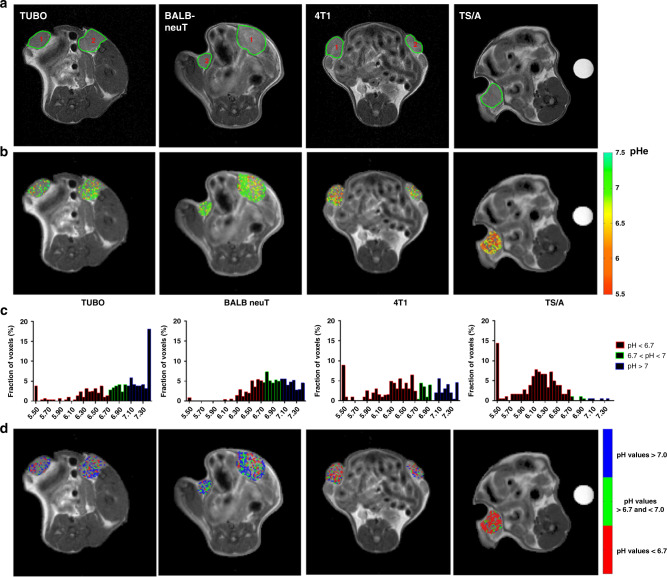
4T1 and TS/A displayed more acidic tumour environment. **a** Anatomical T_2w_ images of TUBO, BALB-neuT, 4T1 and TS/A representative tumours. **b** Representative tumour extracellular pH maps for TUBO, BALB-neuT, 4T1 and TS/A tumours. **c** pH frequency distribution of TUBO, BALB-neuT, 4T1 and TS/A representative tumours. For TUBO, BALB-neuT and 4T1, the distribution was calculated as the mean frequency distribution in the two tumours. **d** Corresponding acidity score maps (colour-coded as red for pixels showing pH values <6.7; green for pH values >6.7 and <7; blue for pH values >7.0) superimposed on anatomical images. Tumour pHe and acidity score values are shown only within tumour regions for improving clarity.

Altered tumour pHe is known to promote several aspects of tumour progression and invasion. We therefore next asked whether the level of tumour acidification may correlate with the formation of lung metastases. MRI-CEST pHe maps were acquired in vivo in tumour-bearing mice upon intravenous administration of iopamidol, a pH-responsive agent. All the investigated murine breast cancer models showed a marked extracellular acidification, with mean pHe values of 6.84 ± 0.03, 6.96 ± 0.03, 6.79 ± 0.02, and 6.8 ± 0.03 for TUBO, BALB-neuT, 4T1, and TS/A, respectively (Fig. 4a). Significant differences were observed between the mean pHe values of the more aggressive breast cancer lines (TS/A and 4T1) and the less aggressive BALB-neuT model (*P* < 0.01 and *P* < 0.05 for TS/A and 4T1, respectively). The corresponding mean acidity score values (Fig. 4b) of the TS/A and 4T1 breast tumours (2.10 ± 0.03 and 2.16 ± 0.03, respectively) were significantly higher than the acidity score values for the TUBO and BALB-neuT ones (1.94 ± 0.05 and 1.92 ± 0.06, respectively). No significant differences were observed within the two aggressive cell lines (TS/A and 4T1), as well as between the two least aggressive models (TUBO and BALB-neuT). To further substantiate these findings, we also evaluated the relationship between tumour acidosis and the metastatic potential. A marked correlation was found between the tumour pHe and the number of lung metastases that was also statistically significant when considering the acidity score (*P* < 0.05, Fig. 4c, d). Representative anatomical MR images of the investigated tumours are shown in Fig. 5a. Figure 5b displays corresponding tumour pHe images of the aggressive (4T1, TS/A) and the indolent (TUBO, BALB-neuT) breast tumours, respectively. Differences in the pHe distribution were observed among the four breast tumour murine models. In Fig. 5c, the frequency distributions of the tumour pHe values are plotted for each group. TUBO and BALB-neuT tumours showed histograms shifted to more neutral values, whereas 4T1 and TS/A were shifted to more acidic values. To further evaluate the heterogeneity of the acidic pHe distribution, the acidity score index was calculated for all the four breast tumour lines. Figure 5d shows representative acidity score images for each breast cancer cell line. The tumours from the less aggressive TUBO and BALB-neuT models exhibited a nearly homogeneous distribution of pixels showing close to neutral pHe values (green-coloured pixels) within the entire tumour, with small regions displaying relatively low pHe values. Conversely, the tumours from the more aggressive TS/A and 4T1 showed heterogeneous distribution of pHe values, with higher fractions of more acidic values (pH < 6.7, red-coloured pixels). ANOVA analysis indicated a significant difference in the acidity scores between the two most and the two least aggressive breast cancer types (*P* = 0.0003).

### Adaptation to chronic acidic pHe induces a stemness phenotype and increases tumour acidosis and lung metastases

Aiming to further study whether acidity may be an aetiological factor in promoting metastasis, we adapted 4T1 cancer cells to a chronic acidic environment, by culturing them in a medium buffered at pH 6.8.^36^ We maintained tumour cells in this medium for 10 weeks until they completely recovered and grew at the same rate as parental cells (Supplementary Fig. S7a). We first investigated the expression of CSC markers and we observed that 4T1 cultured at pH 6.8 expressed a higher level of CD44, CD24 and Sca1 compared to control 4T1 (Supplementary Fig. S7b–d, CD44 1.48 ± 0.76, CD24 1.50 ± 0.42 and Sca1 2.07 ± 0.58 fold increase for 4T1 pH 6.8, ***P = 0.0007), suggesting that cells adapted to an acidic pHe may have a partial CSC phenotype.

Then, we examined in the 4T1 and the 4T1 cultured at pH 6.8 cells the expression of NHE1, CA9 and MCTs transporters by means of quantitative PCR (qPCR) (Supplementary Fig. S7e–h). The qPCR data showed that both cell lines express similar levels of NHE1, whereas CA9 levels were lower in 4T1 cultured at pH 6.8 (0.21 ± 0.08 relative CA9 mRNA expression for 4T1 pH 6.8, *P = 0.03). Conversely, a higher level of both MCT1 and MCT4 mRNA was expressed by 4T1 cells conditioned at pH 6.8 (1.61 ± 0.25 and 3.24 ± 0.60 relative MCT1 and MCT4 mRNA expression for 4T1 pH 6.8, respectively).

However, the invasive potentials of these cells were not influenced by chronic acidic condition, with 4T1 pH 6.8 cells showing similar migration or even reduced invasion capability (Supplementary Fig. S7i, j). A reduced invasive potential for cancer cells acclimated at acidic pH has been already observed for other human breast and melanoma cell lines, in which a selection of a more aggressive phenotype was evident only when re-exposing the adapted cells to a neutral pH.^44^

Tumour developed from the 4T1 pH 6.8 cells, after an initial longer lag-time period of growth (Supplementary Fig. S8), showed a two-fold increase in the number of lung metastases compared to mice injected with 4T1 cells (2.11 ± 0.14 number of lung metastases for 4T1 pH 6.8 relative to 4T1, **P = 0.007, Supplementary Fig. S9c). In vivo tumour pHe imaging showed a more acidic pHe (Supplementary Figs. S9a and S10, 6.83 ± 0.03 and 6.73 ± 0.05 for 4T1 and 4T1 pH 6.8 cells, respectively) and a marked increase of the acidity score although not statistically significant (Supplementary Fig. S9b, 2.04 ± 0.03 and 2.19 ± 0.06 for 4T1 and 4T1 pH 6.8 cells, respectively) for tumour derived from 4T1 cells conditioned to a pH of 6.8 compared to naïve 4T1.

To further explore the link between acidosis and metastatic potential, we manipulated in vivo the pHe of the 4T1 derived tumours by administering sodium bicarbonate to the mice by assuming a reduction in tumour acidity and hence decreased lung metastases.^45^ However, bicarbonate treatment was not effective in increasing tumour pHe and this was associated with the inability to reduce the number of lung metastases generated by 4T1 breast tumour treated with bicarbonate (Supplementary Figs. S10 and S11a–c).

Overall, these data provide a strong evidence that an increased extracellular acidity is associated with cancer invasion and metastasis.

## Discussion

This study aims to validate tumour pHe as an innovative biomarker of metastatic potential in several breast cancer murine models. We have exploited in vivo tumour pHe measurements to characterise three transplantable breast cancer mouse and one transgenic breast tumour models, of which two are aggressive (highly metastatic: 4T1 and TS/A), and two are indolent (poorly metastatic: TUBO and BALB-NeuT). A recognised hallmark of cancer commonly associated to cancer aggressiveness is the abnormally high rate of glycolysis and of lactate production, that is the end product of fermentative glucose metabolism. Therefore, high tumour lactate levels have been correlated with metastasis likelihood in several cancers,^46^ despite other studies found no significant relationships or prognostic capabilities.^47^ On the other hand, tumour acidosis is the cumulative outcome of several processes that are involved in a complex interplay in shaping tumour microenvironment.^48,49^ In fact, acidosis is the result of (i) defective and chaotic vasculature formation, (ii) proton accumulation from several pathways including ATP hydrolysis, pentose phosphate and serine besides glycolysis, (iii) increased expression of proton transporters such as H^+^-ATPases, Na^+^/H^+^ exchangers, Na^+^/HCO3^−^ co-transporters^50,51^ and monocarboxylate transporters and (iv) lactate produced by cells other than tumour cells that sustains the tumour growth and proliferation.^52^ The key role played by tumour acidosis in cancer aggressiveness is further validated by the ability to induce on tumour-associated macrophages and fibroblasts the secretion of cathepsins that degrade the ECM and cell-adhesion molecules promoting invasiveness.^53,54^ In addition, it has been shown that buffering the acidic tumour microenvironment reduces metastasis formation.^45^ Therefore, acidosis may be a superior biomarker for understanding the complex role of the tumour microenvironment in cancer progression.

Only recently, the development of MRI-CEST based pH imaging has provided a powerful tool for a comprehensive in vivo investigation of tumour acidosis.^25^ This approach showed a marked acidic microenvironment for two well-established human breast tumour models, MCF-7 and MDA-MB-231 cells, consistent with a upregulated glycolytic metabolism.^55^ Herein, by mapping in vivo tumour pHe, we show that the more aggressive breast tumours displayed a more pronounced tumour acidic microenvironment, whereas the less aggressive tumours showed a relatively less acidic environment. Although the average tumour acidosis measurements did not provide a clear differentiation between the less and more aggressive tumours models, the exploitation of the acidity score index, which considers the heterogeneity of the tumour acidosis in the whole tumour, allowed to clearly distinguish the two different tumour types.

Several imaging-based studies have investigated different aspects of the tumour microenvironment as potential reporters of the metastatic potential. A fluorescent pH-activatable probe for optical imaging was exploited for assessing differences in acidic microenvironment in two human hepatic xenografts (HepG2 and HCCLM3).^56^ However, although the two tumour models showed a different metastatic potential, in vivo optical images did not provide any clear discrimination, likely due to the low pH sensitivity of the probe and to the inherently limited tissue depth penetration of the technique. Recently, ex vivo analysis by exploiting a fluorescent pH-responsive probes showed that tumour acidic regions overlap with highly proliferative and invasive regions at the tumour–stroma interface, likely promoting tumour invasion.^57^ Positron emission tomography-based tracers targeting tumour acidosis^58^ or for imaging tumour hypoxia or for targeting CXCR4 chemokine receptor involved in metastasis^59^ have been proposed with promising clinical translatability, despite radiation exposure and limited spatial resolution hamper accurate heterogeneity measurements. Tumour vessel permeability has been investigated on xenograft models of metastatic and non-metastatic breast and prostate tumour using MRI albumin-GdDTPA contrast agent, providing evidence that an adequate vascularisation and/or permeability is needed for metastasis to occur.^12^ Overall, these studies highlight the importance of MRI-based methods that can provide sufficient spatial resolution, depth penetration and pH accuracy for non-invasively assessing tumour pHe and for accurate quantification of its heterogeneity. Despite the higher accuracy in assessing extracellular tumour pH for MRI-based methods that require the injection of a pH-responsive probe, the low extravasation of the agent in poorly vascularised or necrotic tumour areas can hinder the pH measurement in these regions. Anyway, in the investigated tumour murine models, the high accumulation of the pH-responsive probe in the whole tumours (as indicated by the high coverage of coloured pixels in the tumour regions) resulted in pH measurements representative of the whole tumour. Moreover, the large heterogeneity that we observed in tumour pHe values among the investigated tumour murine models is in agreement with the range of pHe values reported by others using MRI-based approaches and confirmed by invasive microelectrode measurements.^60–63^

Importantly, the relationship between tumour acidosis and metastatic propensity is a valuable clinical issue, since death rate in breast cancer patients is heavily dependent on metastases. In addition to the strong correlation observed between the acidity score and the number of lung metastases, we further investigated whether a causative link exists between tumour acidosis and invasiveness. First, the acclimation of the 4T1 tumour cells to low pH promoted CSC markers and increased expression levels of MCT1/4 proton transporters that resulted, when inoculated in mice, in an increased tumour acidic microenvironment and in a two-fold increase of lung metastases. Second, prolonged bicarbonate treatment was not able to induce any change in tumour acidosis and this was accompanied by similar numbers of lung metastases. Although bicarbonate treatment was initially proposed to reduce the incidence of metastases by inhibiting tumour acidity, several studies have shown contradictory results with a strong dependence on the investigated tumour types.^45,64^ Our in vivo data support this hypothesis of a causative link between tumour acidosis and metastatic potential, but it is still not possible to draw a strong conclusion. Therefore, new studies will be required to investigate whether the reduction of tumour acidosis will hamper and delay the development of metastases. Furthermore, since metabolic pathways essential for tumour growth are being explored as novel targets to anti-cancer drug development, accurate in vivo pH imaging may be a promising tool for monitoring and predicting the therapeutic response.^65^

In conclusion, we demonstrate that more aggressive breast cancer cell lines present more acidic tumour pHe that results in increased lung metastases. Our data suggest that MRI-CEST imaging of tumour pHe may be a valuable tool for predicting the metastatic potential of primary tumours. However, additional studies are necessary to verify the generalisability of these findings in other cancer types. These results may open new interest in assessing the role of tumour acidosis in several aspects of tumour progression and response to therapy.

## Supplementary information

Supplementary Materials

## Data Availability

The data that support the findings of our study are available from the corresponding author upon reasonable request.
