# Supplementary material for: Tumour acidosis evaluated in vivo by MRI-CEST pH imaging reveals breast cancer metastatic potential
Source: Br J Cancer. 2020 Dec 1;124(1):207–16. doi: 10.1038/s41416-020-01173-0 (PMC7782702; doi:10.1038/s41416-020-01173-0)
Supplement: Supplementary file 1 — Supplementary Materials [file 41416_2020_1173_MOESM1_ESM.docx]

**Tumour acidosis evaluated in vivo by MRI-CEST pH imaging reveals breast cancer metastatic potential**

Annasofia Anemone^1^, Lorena Consolino^1^, Laura Conti^2^, Pietro Irrera^3^, Myriam Hsu^1^, Daisy Villano^1^, Walter Dastrù^1^, Paolo Porporato^2^, Federica Cavallo^2^ and Dario Livio Longo^4*^

^1^ Molecular Imaging Center, Department of Molecular Biotechnology and Health Sciences, University of Torino, Via Nizza 52, Torino, Italy

^2^ Department of Molecular Biotechnology and Health Sciences, University of Torino, Via Nizza 52, Torino, Italy

^3^ University of Campania “Luigi Vanvitelli”, Caserta, Italy

^4^ Institute of Biostructures and Bioimaging (IBB), Italian National Research Council (CNR), Via Nizza 52, Torino, Italy

**SUPPLEMENTARY FILES**

**Supplementary Methods**

**Cell migration and invasion assays**

TUBO, 4T1 and TS/A cell lines were seeded in 6-well plates and grown to at least 95% of confluent monolayers as reported in literature [^1^](#_ENREF_1). Cells were then scratched with a 200µL tip to make wounds. The cells were then rinsed with PBS to remove the loosened cell debris. Culture medium was added to the cells and the plates were incubated at 37°C in 5% CO_2_ for 24h and 48h. The wound was observed using phase contrast microscopy. As the cells migrated to fill the scratched area, images were captured and the distance between the edges was calculated using ImageJ software. The extent of wound closure was determined as: wound closure (%) = 1 - (wound width t_x_ / wound width t_0_) x 100. All experiments were performed at least three time.

To evaluate the invasive activity, 24-well trans-well chambers (Corning, NY) containing inserts with polycarbonate membranes with 8µm pores coated with 50µL of diluted (1:3 in culture medium) Matrigel (Corning, NY) were used. TUBO, 4T1 and TS/A cells were harvested, suspended in serum-free medium and counted. Cells (1x10^5^ in 100 µL serum-free medium per well) were then added to each top chamber. In the lower well medium containing 10% of FBS as chemoattractant was added. After 24 and 48 h of incubation at 37°C in a humidified incubator in 5% of CO_2_, the non-invading cells were removed using a cotton-tip, and the cells migrated to the lower surface of the membrane were fixed in 70% ethanol and stained with 0.2% crystal violet (Sigma-Aldrich, St.Louis, MO, USA). Using a light microscope, the total number of stained cells was determined from five randomized fields.

**RNA extraction and** **real-time PCR**

RNA was isolated from fully confluent cells with trizol (Invitrogen) and retro-transcribed to cDNA using high capacity reverse transcription kit (Life technologies). Transcript levels were determined using SYBR green PCR master mix (Applied Biosystems) with QuantStudio 6 real-time instrument (Applied Biosystems) according to the manufacturers. The following primer sequences were used: RPL19 ATGAGTATGCTCAGGCTACAGA (forward); GCATTGGCGATTTCATTGGTC (reverse), MCT1 GTGCAACGACCAGTGAAGTATC (forward); CGATCAAGCCGCAACCAGA (reverse), MCT4 GGACGTCGAGACCCATTTAG (forward); GGCGACGCTTGTTGAAGTAT (reverse), CA9 TGCTCCAAGTGTCTGCTCAG (forward); CAGGTGCATCCTCTTCACTGG (reverse), NHE1 CACCCTTTGAGATCTCCCTCT (forward); GGGGATCACATGGAAACCTA (reverse). All the transcripts are expressed as fold change normalized to RPL19 transcript and compared to values of TUBO cells or to 4T1 when compared to 4T1 conditioned to pH 6.8.

**Extracellular pH measurement**

A total of 5 × 10^4^ TUBO, TS/A or 4T1 cells were seeded into 96-well plates using their own culture media and incubated overnight to allow adherence in a standard CO_2_ incubator. The next day pH measurements were performed with the pH-Xtra Glycolysis Assay (Luxcel Bioscience, Cork, Ireland) kit including Respiration Buffer (RB) and Fluorescent Probe (FP) using a microplate reader (BioTek Instruments, Inc., Winooski, VT, USA). Following the manufacturer protocol, 96-well plates were removed from standard incubator (5% CO_2_, 37 °C) and maintained in a CO_2_-free incubator at 37 °C for two hours allowing the gas to purge. After that, growth medium was removed and each well washed twice with respiration buffer (RB) and then filled with the FP-RB solution (1:10, total volume per well 100 uL). Prior to its use, RB tablet was resuspended in 50 mL bi-distilled water titrated to pH 7.4 with NaOH 1M and then filtered with 0.2 µm filter whereas the FP was resuspended in 1 mL bi-distilled water. Fluorescence intensity was measured kinetically for two hours (data not shown) ending with a dual-read time-resolved fluorescence mode keeping the reader at 37 °C. This dual-read mode takes into account the fluorescence decay measuring fluorescence intensity with two different delays for data collection allowing the calculation of the lifetime (µs) for each well with the formula [D_2_-D_1_/ln(R_1_/R_2_)] where D_1-2_: delay 1=100 µs, delay 2=300 µs; R_1-2_: read 1, read 2. Finally, is possible to derive pH values using lifetime values in the calibration curve (previously calculated titrating RB at different pH and performing the dual-read) formula pH= a (lifetime) + c.

RB composition: 1 mM K-phosphate, 20 mM Glucose, 70 mM NaCl, 50 mM KCl, 0.8 mM MgSO_4_, 2.4 mM CaCl_2._

FP composition: pH-sensitive fluorescent lanthanide probe.

***In vivo* MRI-Chemical Exchange Saturation Transfer pH imaging and analysis**

MR-CEST images were acquired on a Bruker Avance 7T MRI scanner using a 30mm insert coil. Z-spectra were acquired using a single-shot RARE sequence with centric encoding (typical setting TR/TE/NEX = 6.0 s/4.14 ms/1) preceded by a 3µT cw block presaturation pulse for 5 s and by a fat-suppression module. A series of 40 MR frequencies were saturated to acquire a CEST spectrum in the frequency offset range ±10 ppm. We used an acquisition matrix of 96x96 reconstructed to 128x128 for a field of view of 3x3 cm^2^ (in-plane spatial resolution = 234 µm) with a slice thickness = 1.5 mm. MR-CEST images were repeated eight times, once before and seven times after i.v. injection of the iodinated contrast media (dose = 4 g Iodine / kg body weight). The total scan time was 32 min.

All CEST images were analyzed using a home-made script implemented in MATLAB (The Mathworks, Inc., Natick, MA, USA). The Z-spectra were interpolated, on a voxel-by-voxel basis, by smoothing splines, B_0_-shift corrected and saturation transfer efficiency (ST%) was measured by punctual analysis [^2^](#_ENREF_2). For in vivo images, difference contrast maps (ΔST%) were calculated by subtracting the ST contrast after iodinated contrast media injection from the ST contrast before the injection on a per voxel basis in order to reduce the confounding effect of the endogenous contributions. A threshold value of 1% was set to discriminate between enhancing and not-enhancing pixels. pHe values were estimated in vivo from CEST images 15 min after the Iopamidol injection, applying the ratiometric procedure [^3^](#_ENREF_3). The pHe maps were superimposed onto the anatomical reference image. pH values variation was represented in terms of frequency distributions. Pixels with pH values lower than 5.5 or greater than 7.4 pH units were assigned to 5.5 or 7.4, respectively.

**FACS analysis**

For flow cytometry-based glucose uptake assays, 4x10^5^ cells were incubated at 37°C with 100 μM 2-NBDG (2-deoxy-2-[(7-nitro-2,1,3-benzoxadiazol-4-yl) amino]-d-glucose, Focus biomolecules, Plymouth, USA) dissolved in a glucose-free medium for 10 minutes, and their fluorescence measured by flow cytometry as previously described [^4^](#_ENREF_4).

After 24h of culture, TUBO, 4T1 and TS/A cells were collected and disaggregated using enzymatic and mechanical dissociation. Then they were washed in PBS and stained for membrane antigens. The following antibodies were used: (i) Alexa Fluor647-conjugated anti-Stem Cell Antigen-1 (Sca-1), (ii) PE-conjugated anti-CD44 and (iii) PE/Cy7-conjugated anti-CD24 (all from Biolegend), as reported [^5^](#_ENREF_5).

All samples were collected and analyzed using a CyAn ADP Flow Cytometer and Summit 4.3 software (DakoCytomation).

For phenotypic analysis, fresh primary tumours of 10 mm mean diameter (400mm^3^) from BALB-neuT mice or from BALB/c mice injected s.c. with TUBO, 4T1 and TS/A cells were processed as reported in *Macagno et al* [*^6^*](#_ENREF_6). Briefly, tumors were finely minced with scissors and then digested by incubation with 1 mg/ml collagenase IV (Sigma Aldrich) in RPMI-1640 (Life Technologies) at 37° C for 1 h in an orbital shaker. After washing in PBS supplemented with 2% FBS, the cell suspension was incubated in an erylise buffer (155mM NH_4_Cl, 15.8mM Na_2_CO_3_, 1mM EDTA, pH 7.3) for 10 minutes at RT. After washing in RPMI-1640 supplemented with 10% FBS, the cell suspension was passed through a 40-µm pore cell strainer and centrifuged at 1400 rpm for 10 minutes. 1 x 10^6^ cells re-suspended in PBS were treated with Fc receptor blocker (anti-CD16/CD32; BD Biosciences), and stained with VioGreen-anti-CD45 (Miltenyi Biotec), AlexaFluor647-anti-Sca-1, PE-anti-CD44 and PE/Cy7-anti-CD24 antibodies (Biolegend) for 30 min at 4° C, as in Lanzardo S et al [^7^](#_ENREF_7). Samples were analyzed on a CyAnADP Flow Cytometer, using Summit 4.3 software (Beckman Coulter). Analysis was conducted on CD45- tumour cells, to exclude immune infiltrating cells.

**Immunofluorescence microscopy**

Cells grown on glass coverslips for 24 h were fixed with cold methanol and permeabilized. After blockade, cells were incubated for 1 h RT with the primary antibody (GLUT1, Abcam, 1:100), for 1h with the secondary antibody (1:500 anti-rabbit Alexa Fluor 488, Invitrogen) and then stained with DAPI. Coverslips were then mounted using the fluorescence mounting medium (ProLong, Life Technologies). For each cell line glass, we considered six cells and six background area to normalize the fluorescence intensity values.

Frozen tumor slices (5-μm thickness) were fixed with cold acetone for 10 min and dried in air for 30 min. The slices were rinsed with PBS and blocked with 10% goat serum for 30 min RT. The slices were then incubated with rabbit anti-mouse antibody overnight at 4°C and visualized using anti-rabbit secondary antibody (1:500; anti-rabbit Alexa Fluor 488, Invitrogen).

All the immunofluorescence stainings were analyzed using an ApoTome system microscope (Zeiss) and the fluorescent signal was quantified using the ImageJ software; the fluorescence intensity values were then normalized to the background fluorescence for each field.

**Supplementary Figures**

**Figure S1**: Representative density plots showing the presence of Sca-1^+^, or CD44^+^ CD24^-^ expressing cells from 3 independent experiments.

**Figure S2:** Representative FACS histograms showing 2-NBDG uptake (gray) and the control cell without 2-NBDG (open histograms).

**Figure S3:** (a and b) Protein expression level of CA9 and NHE1detected by western blot analysis. Vinculin was used as a loading control. Signal quantification was expressed compared to TUBO cells. (c-d) mRNA expression level of MCT1 and MCT4 detected by qPCR. Signal quantification was expressed compared to TUBO cells.

**Figure S4**: Column-bar graph showing the area occupied by metastases compared to the whole lung area of BALB/c mice s.c injected with TUBO, 4T1 or TS/A cells and of BALB-neuT mice

**Figure S5:** Fluorescence microscopy images of tumor TUBO, BALB neuT, 4T1 and TS/A slice stained for DAPI, GLUT1 and merge images, white bar (10µm).

**Figure S6:** Representative density plots showing the expression of Sca-1, or of CD44 and CD24 in 10 mm mean diameter tumours (400mm^3^) explanted from BALB-neuT mice or from BALB/c s.c. injected with TUBO, 4T1 or TS/A cells from 3 independent experiments.

**Figure S7**: (a) Growing curve of 4T1 and 4T1 pH6.8 cell lines. (b-d) Graph showing the expression of Sca-1, CD44 and CD24 in cells from 3 independent experiments (fold increase compared to 4T1 cells). (e-f) mRNA expression level of NHE1, CA9, MCT1 and MCT4 detected by qPCR. Signal quantification was expressed compared to 4T1 cells. (i-j) wound repair capability and invasion capability of 4T1 and 4T1 pH6.8 cell line.

**Figure S8:** Growing curve of 4T1 and 4T1 pH6.8 tumours inoculated in female BALB/c mice.

**Figure S9:** (a and b) Mean extracellular pH and acidity score calculated by MRI CEST pH imaging for 4T1 and 4T1 adapted to pH 6.8 mice. (c) Relative number of lung metastases calculated for mice injected with 4T1 or 4T1 adapted to pH 6.8 cells.

**Figure S10**: (a) Anatomical T_2w_ images of 4T1, 4T1 pH6.8 and 4T1 treated with sodium bicarbonate representative tumours. (b) Representative tumour extracellular pH maps for 4T1, 4T1 pH6.8 and 4T1 treated with sodium bicarbonate tumours. (c) Corresponding acidity score maps (color-coded as red for pixels showing pH values <6.7; green for pH values >6.7 and <7; blue for pH values >7.0) superimposed on anatomical images. Tumour pHe and acidity score values are shown only within tumour regions for improving clarity.

**Figure S11**: (a and b) Mean extracellular pH and acidity score calculated by MRI CEST pH imaging for 4T1 and 4T1 treated with 200mM of sodium bicarbonate mice. (c) Relative number of lung metastases calculated for mice injected with 4T1 and 4T1 treated or not with 200mM of sodium bicarbonate mice.

**Figure S1**


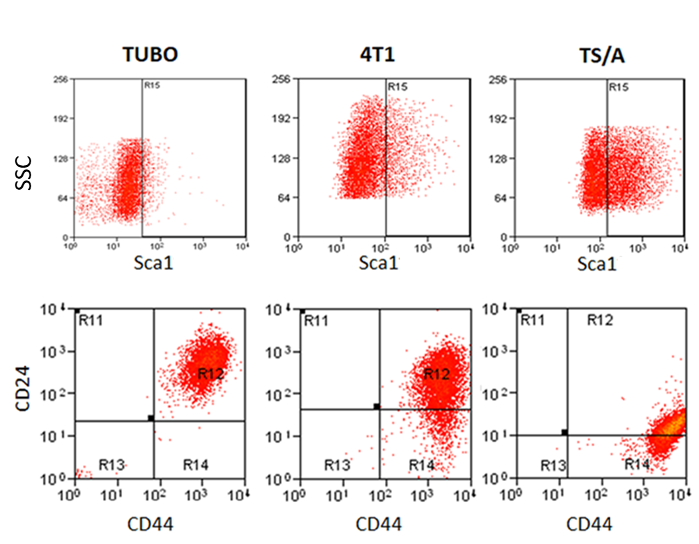


**Figure S2**


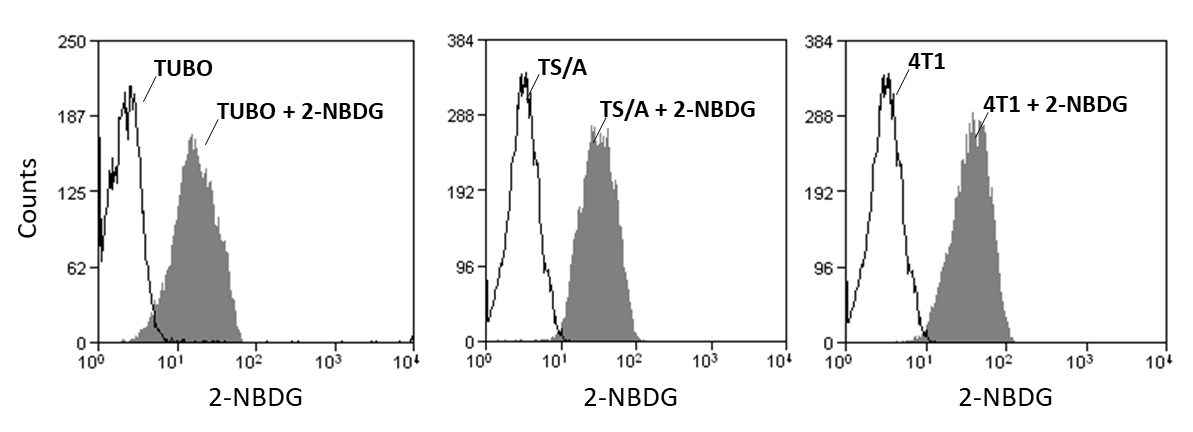


**Figure S3**


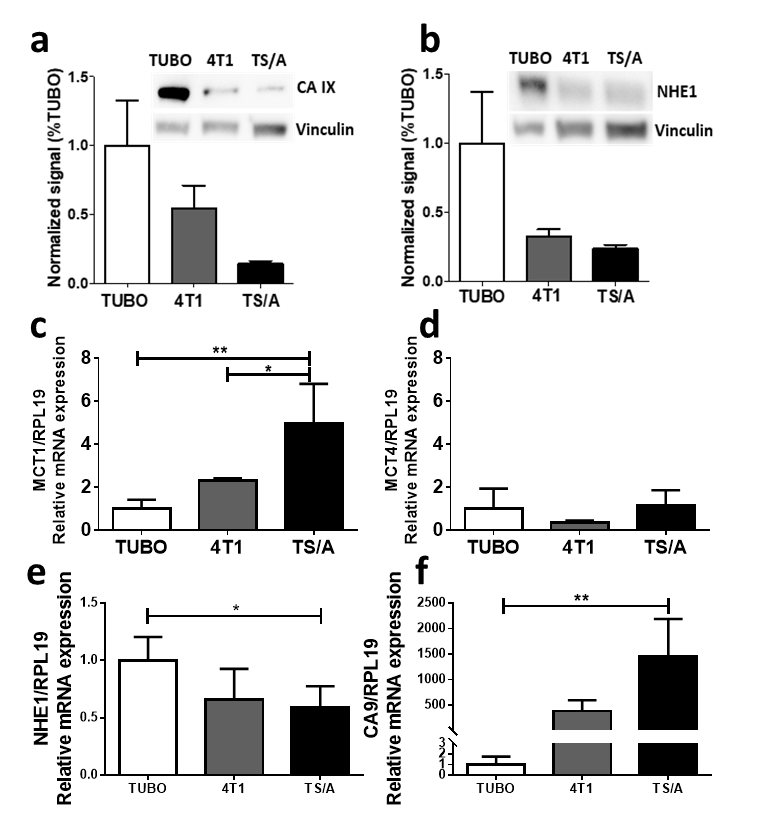


**Figure S4**

**
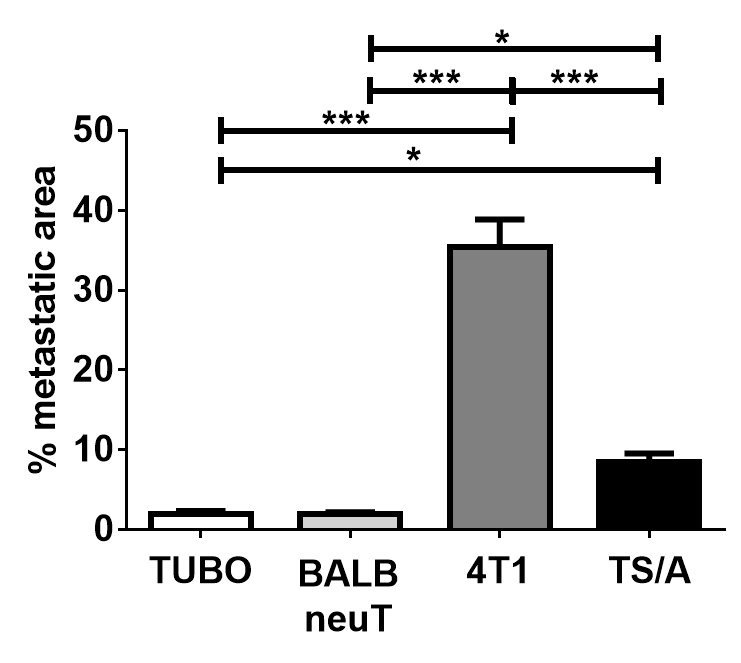
**

**Figure S5**


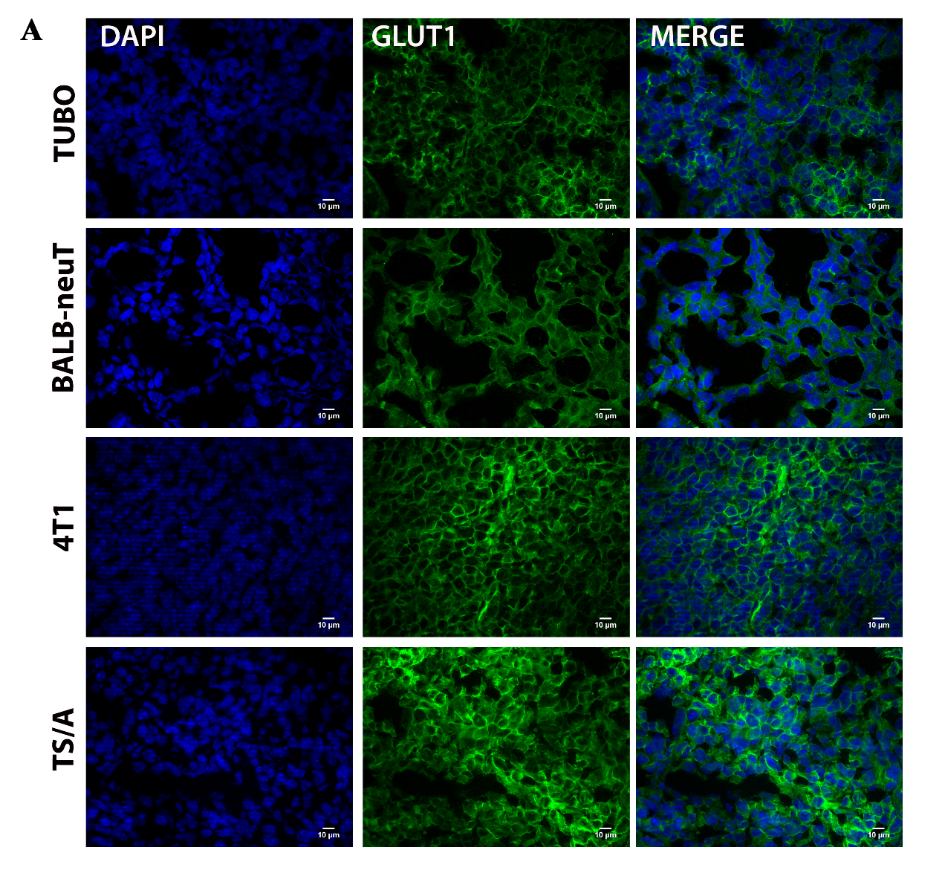


**Figure S6**


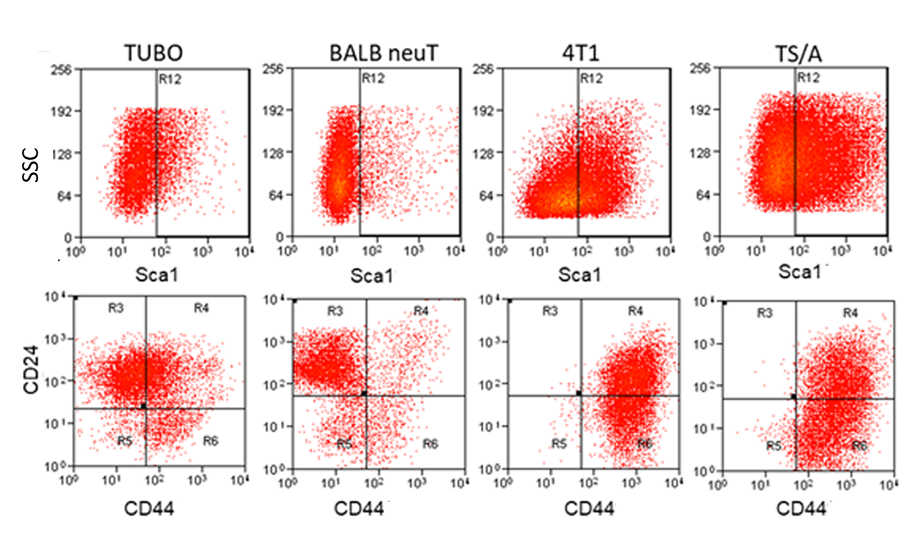


**Figure S7
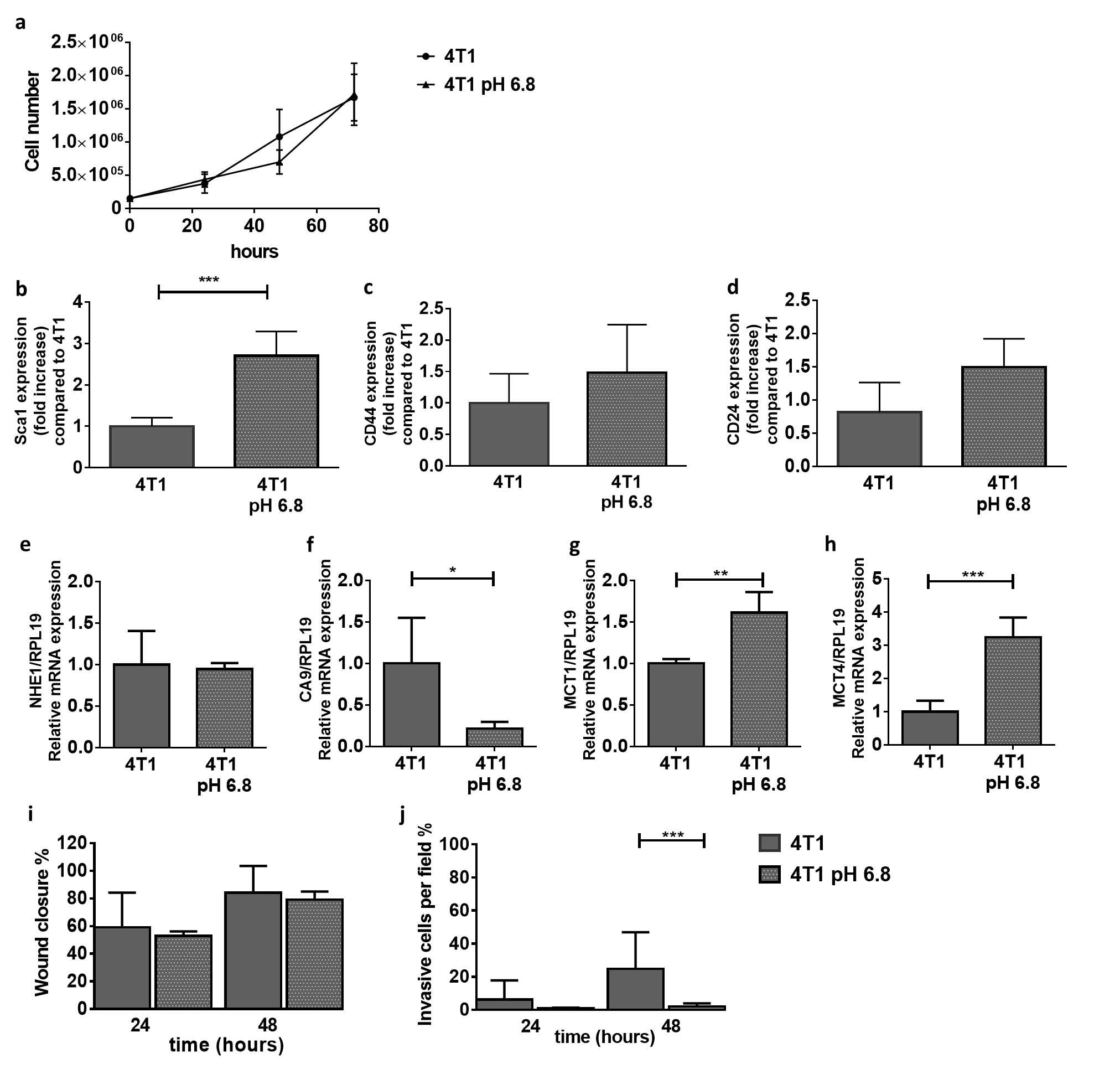
**

**Figure S8**

**Figure S9**

**
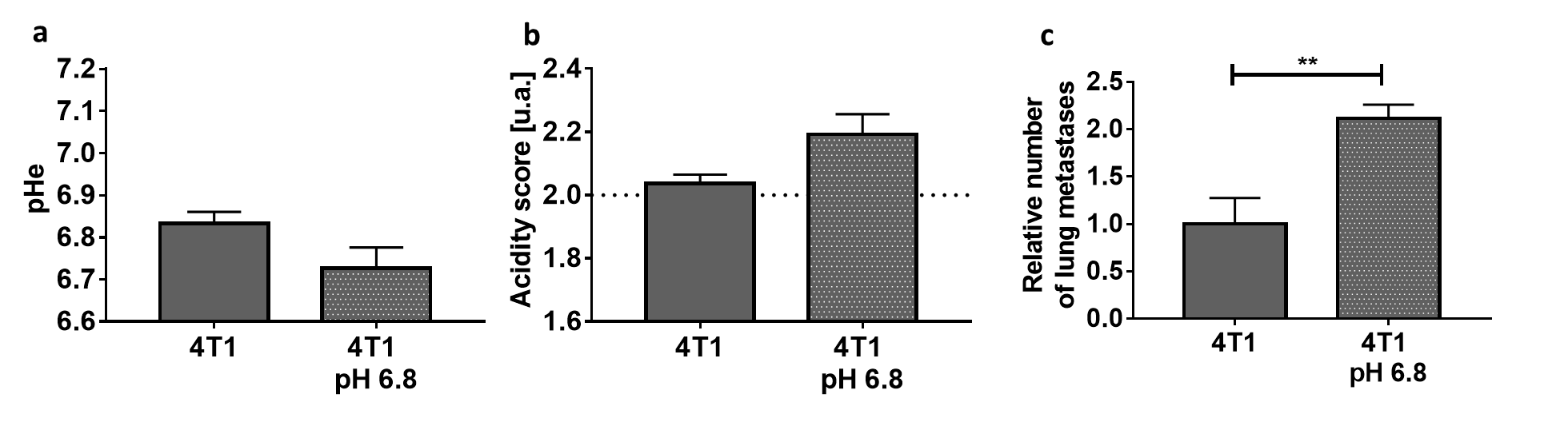
**

**Figure S10
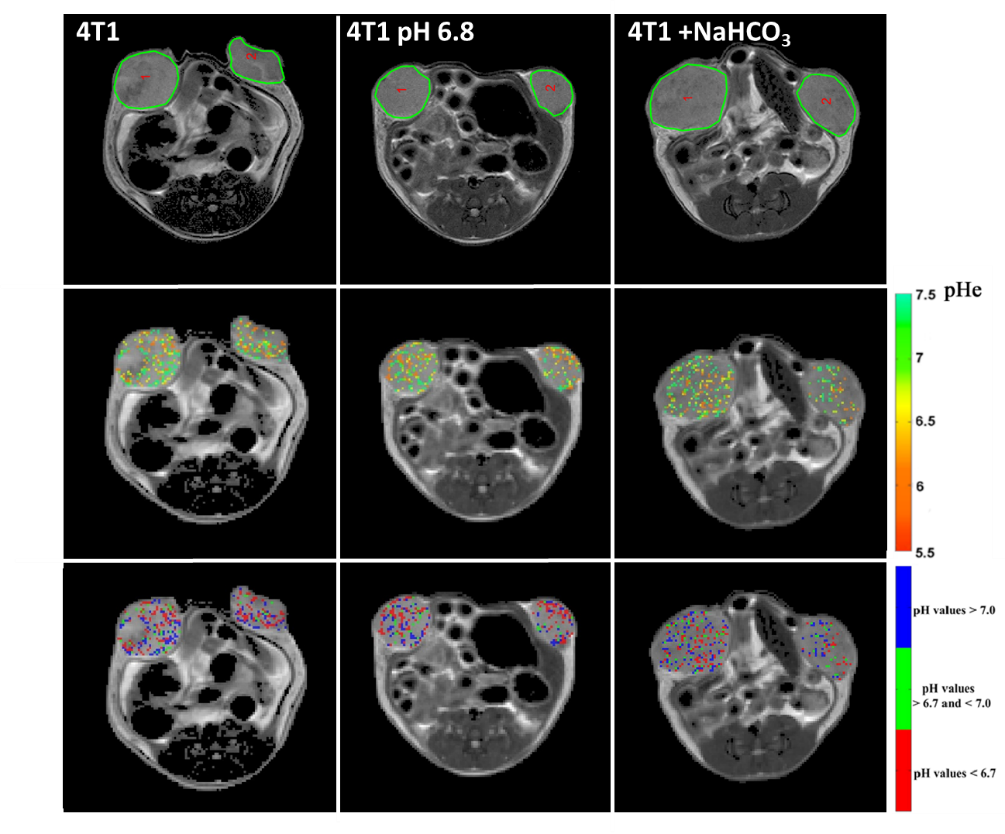
**

**Figure S11**

**
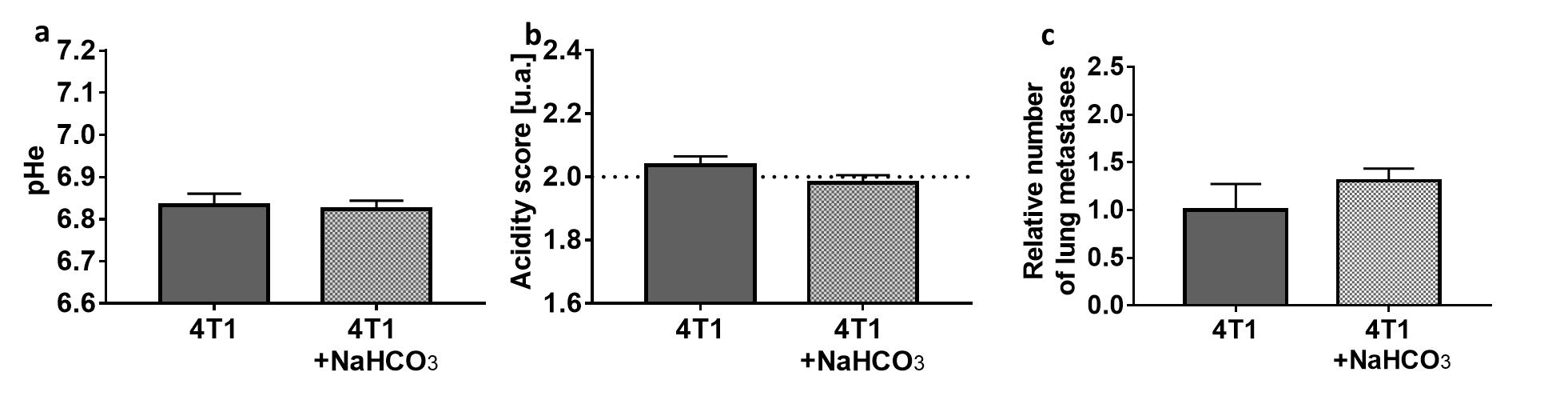
**

**References**

1. Justus CR, Leffler N, Ruiz-Echevarria M, Yang LV. In vitro cell migration and invasion assays. Journal of visualized experiments : JoVE. 2014(88).

2. Terreno E, Stancanello J, Longo D, Castelli DD, Milone L, Sanders HM, et al. Methods for an improved detection of the MRI-CEST effect. Contrast Media Mol Imaging. 2009;4(5):237-47.

3. Sun PZ, Longo DL, Hu W, Xiao G, Wu RH. Quantification of iopamidol multi-site chemical exchange properties for ratiometric chemical exchange saturation transfer (CEST) imaging of pH. Physics in Medicine and Biology. 2014;59(16):4493-504.

4. Zou C, Wang Y, Shen Z. 2-NBDG as a fluorescent indicator for direct glucose uptake measurement. Journal of biochemical and biophysical methods. 2005;64(3):207-15.

5. Fornari C, Beccuti M, Lanzardo S, Conti L, Balbo G, Cavallo F, et al. A mathematical-biological joint effort to investigate the tumor-initiating ability of Cancer Stem Cells. PloS one. 2014;9(9):e106193.

6. Macagno M, Bandini S, Stramucci L, Quaglino E, Conti L, Balmas E, et al. Multiple roles of perforin in hampering ERBB-2 (Her-2/neu) carcinogenesis in transgenic male mice. Journal of immunology. 2014;192(11):5434-41.

7. Lanzardo S, Conti L, Rooke R, Ruiu R, Accart N, Bolli E, et al. Immunotargeting of Antigen xCT Attenuates Stem-like Cell Behavior and Metastatic Progression in Breast Cancer. Cancer research. 2016;76(1):62-72.
